# Aprotinin in high-risk isolated coronary artery bypass graft patients: a 3-year propensity matched study

**DOI:** 10.1186/s13019-024-02837-1

**Published:** 2024-07-18

**Authors:** Rishab Makam, Ayush Balaji, Marwan Al Munaer, Shantanu Bajaj, Nabil Hussein, Mahmoud Loubani

**Affiliations:** 1 Department of Cardiothoracic Surgery, Castle Hill Hospital, Hull University Teaching Hospitals Trust, Cottingham, UK; 2 University of York, York, UK; 3 Hull Royal Infirmary, Hull University Teaching Hospitals Trust, Hull, UK

**Keywords:** Aprotinin, Isolated coronary artery bypass graft, High-risk surgery, Anti-fibrinolytics, Bleeding, Transfusion, Mortality

## Abstract

**Background:**

Aprotinin, a serine protease inhibitor, has been used variably in cardiac surgery amidst ongoing debates about its safety following several previous studies. This study assesses the outcomes of aprotinin in high-risk isolated Coronary Artery Bypass Graft (iCABG) patients.

**Methods:**

The study retrospectively analysed a cohort of 1026 iCABG patients, including 51 patients who underwent aprotinin treatment. Logistic regression powered score matching was employed to compare aprotinin patients with a control group, in a propensity-matched cohort of 96 patients. The primary outcome measured was in-hospital death, with secondary outcomes including renal dysfunction, stroke, myocardial infarction, re-exploration for bleeding or tamponade, and postoperative stay durations.

**Results:**

The aprotinin cohort had high-risk preoperative patients with significantly higher EUROSCORE II values, 7.5 (± 4.2), compared to 3.9 (± 2.5) in control group. However, aprotinin group showed no statistically significant increase (p-value: 0.44) in hospital mortality with OR 2.5 [95% CI 0.51, 12.3]. Major secondary outcome rates of renal replacement therapy and postoperative stroke compared to the control group were also statistically insignificant between the two groups.

**Conclusion:**

This study suggests that aprotinin may be safely used in a select group of high-risk iCABG patients. The reintroduction of aprotinin under specific conditions reflects its potential benefits in managing bleeding in high-risk surgeries, but also underscores the complexity of its risk-benefit profile in such critical care settings. Nonetheless, it highlights the importance of carefully selecting patients and conducting additional research, including larger and more controlled studies to fully comprehend the potential risks and benefits of aprotinin.

**Supplementary Information:**

The online version contains supplementary material available at 10.1186/s13019-024-02837-1.

## Introduction

Aprotinin is a nonspecific serine protease inhibitor that acts as an antifibrinolytic agent to prevent the breakdown of clots through the inactivation of plasmin, the preservation of platelet function, and the counteraction of dysregulated activity within the coagulation system. Although it was first discovered in the 1930s [1, 2], its use in major surgery started in the 1960s [3], and its use in cardiac surgery became prevalent only after a 1987 trial showed that the control group had a transfusion requirement eight times greater than the aprotinin group [4].

However, between 2006 and 2008, studies began to show an association between the use of aprotinin and poor postoperative outcomes. Three distinct large observational studies showed renal dysfunction as well as increased mortality with aprotinin use compared with lysine analogues [5–8]. The largest factor was the BART study, a randomised controlled trial comparing aprotinin with tranexamic acid and aminocaproic acid, which showed higher mortality despite reduced bleeding in the aprotinin group [9]. These studies ultimately halted the use of aprotinin by introducing a licence suspension, issuing guideline changes, and warnings regarding adverse effects.

In 2011, Health Canada announced the lifting of the suspension of aprotinin in clinical practice [10]. The European Medicines Agency (EMA) soon followed in 2012 by reintroducing aprotinin use in Europe [11]. Although they each provide specific arguments for reapproval, the prevailing sentiment was concern regarding the reliability of evidence that triggered the suspension of aprotinin. The renewal came with recommendations to reserve aprotinin for high-risk isolated CABG (iCABG) surgery. Additionally, the new guidelines advised better activated clotting time (ACT) monitoring and the establishment of a registry to monitor aprotinin use [12].

Aprotinin was reintroduced at the study centre in 2019 after a thorough evaluation of the literature surrounding its safety was performed. A robust, evidence-based standard operating procedure (SOP) was used to advise the use of aprotinin in high-risk iCABG patients. This study examined the use of aprotinin and associated outcomes in this subset of high-risk patients.

## Methods

### Study design and population

This was a retrospective, observational, cohort study of prospectively collected data from consecutive patients who underwent CABG at a single centre. Preoperative, intraoperative, and postoperative data for all iCABG patients were collected from August 2019 to June 2023. A total of 1245 patients underwent isolated surgical revascularisation since the reintroduction of aprotinin at the centre. All patients were operated on cardiopulmonary bypass.

### Standard operating procedure

**Fig. 1:**
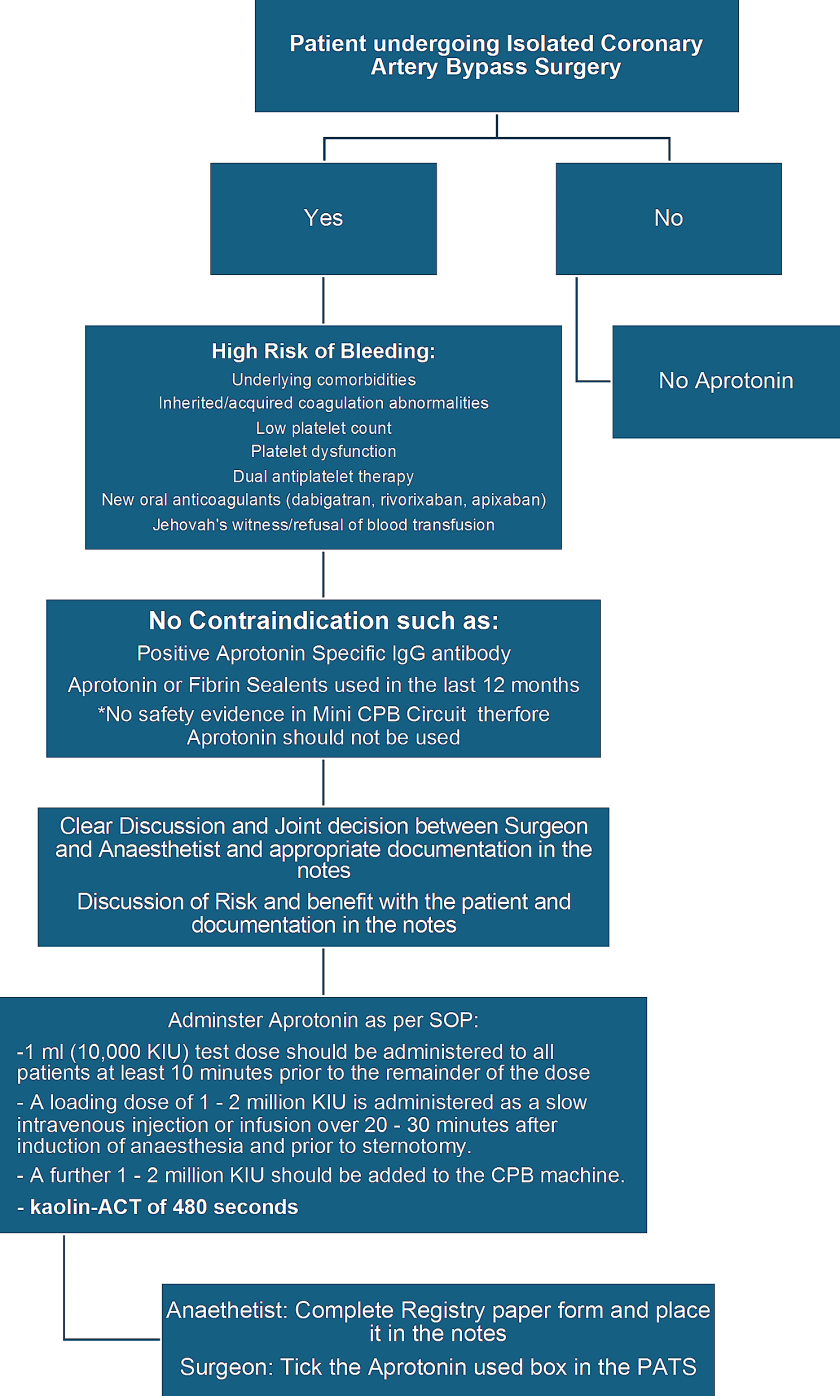
Flowchart of the standard operating procedure for aprotinin use

As per the SOP at the study centre, aprotinin was administered only to patients who were at high risk for blood loss during coronary bypass, as indicated by coagulation abnormalities, low platelet counts (< 150), platelet dysfunction, refusal to transfusion or perioperative anticoagulant or antiplatelet (excluding aspirin) use. Aprotinin was also used in emergency patients, such as coronary dissection patients who required immediate surgery, and in urgent patients when anticoagulation or dual antiplatelet therapy (DAPT) could not be discontinued prior to surgery. Patients with other underlying comorbidities such as severe kidney or liver disease, nutritional deficiencies, and poorly controlled inflammatory diseases also received aprotinin treatment. Figure 1 shows a simplified flowchart of the SOP but further information regarding the complete SOP for aprotinin use in the study centre is included in the supplementary material.

Elderly patients, or those with pre-existing renal or hepatic impairment were treated with the standard regime because there is no statistically significant evidence to support the need for dose adjustments or alternate regimens in these patients. Careful consideration of the risks and benefits for each patient following discussion with the operative team and haematologists was standard protocol prior to aprotinin use. Any deviation from the standard operating procedure was fully discussed and agreed upon between the operating surgeon and anaesthetist.

### Statistical analysis

Extensive data was collected on patient demographics, medical and surgical history, preoperative status, operative data, and postoperative outcomes. A total of 262 data fields for 1244 patients were collected for use in the analysis. The baseline data was recoded, categorised, and converted to binary or ordinal variables to improve their utility in the statistical analysis.

This database was refined to exclude fields and cases for which a significant proportion of the data was incomplete before preparing for statistical analysis. The baseline preoperative variables used in the propensity score model were compiled and compared between the aprotinin group and the control group. Categorical variables were compared using the 2-tailed chi-square test or Fisher’s exact test. Ordinal variables were compared using the Mann‒Whitney U test, and continuous variables were compared using the independent samples t-test. All reported p values were two-sided, and a p value less than 0.05 was used to indicate statistical significance.

A logistic regression model was then created to estimate the probability of intraoperative aprotinin utilisation. This model was subsequently used for propensity score matching to mitigate baseline heterogeneity between the control and aprotinin groups and improve the comparability of postoperative outcomes.

### Propensity matching

Seventy-two of the data points corresponded to preoperative factors that could potentially impact postoperative outcomes. Variables that were redundant, irrelevant, nonsignificant or had a significant proportion of missing data were removed. In total, 24 variables were retained in the logistic regression model for propensity score matching. The model was run to generate a propensity score based on preoperative factors for aprotinin use during surgery. The propensity scores were subsequently used to generate matched subcohorts of control and aprotinin patients for outcome comparison. The subgroups were matched at a 1:1 ratio and constructed using the nearest neighbour matching strategy.

### Outcomes

The primary outcome of the study evaluating the safety of aprotinin was in-hospital death. The secondary outcomes were postoperative red cell transfusion, renal dysfunction requiring new renal replacement, new stroke, postoperative myocardial infarction (MI), re-exploration for bleeding or tamponade, postoperative stay in ICU (hours) and total postoperative stay prior to discharge (days).

## Results

Between August 2019 and June 2023, a total of 1244 patients underwent isolated coronary revascularisation at our centre. After recoding and refinement, 218 patients were removed from the database due to corrupted or incomplete data. Of the 1026 patients included in the data analysis, 51 received aprotinin treatment at the time of surgery. Logistic regression modelling and propensity score matching was conducted for this cohort to mitigate disparities in baseline characteristics between the control and aprotinin-treated patients. After propensity matching, both cohorts contained 48 patients each. The lowest score calliper that allowed a 1:1 matched subgroup was 0.2. However, the calculated c-statistic for our regression model was 0.880, indicating an appropriately discriminatory model. A summary of the 24 baseline preoperative variables that were integrated into the regression model and the resulting propensity scores for the full cohort are reported in Table 1. We have also included the cumulative bypass time (minutes) and cross-clamp time (minutes), although these were not included in the logistic regression as they cannot be considered significant predictors of aprotinin use. The odds ratios for statistically significant differences are reported.

**Table 1:** Baseline patient characteristics (Unmatched cohort)

Baseline characteristics of patients				
Variable	Aprotinin (*N* = 51)	Control (*N* = 975)	*P* value	Odds ratio for aprotinin (95% CI)
**Mean Age (± SD)**	66.1 (± 9.0)	65.9 (± 9.0)	0.88	
**Sex (%)** *Male* *Female*	42 (82.4) 9 (17.6)	815 (83.6) 160 (16.4)	0.82	[Female] 1.08 (0.59,2.0)
**BMI (kg/m** ^**2**^ **) (± SD)**	29.2 (± 4.6) [*N* = 50]	29.7 (± 5.0)	0.46	
**Smoking History (%)** *Never* *Ex-Smoker* *Current Smoker*	13 (25.5) 28 (54.9) 10 (19.6)	363 (37.2) 472 (48.4) 140 (14.4)	0.21	
**Family History of Heart Disease (%)**	27 (56.3) [*N* = 48]	640 (66.0) [*N* = 970]	0.17	0.85 (0.66, 1.1)
**Angina (%)**	51 (100)	962 (98.7)	0.10	1.05 (1.04, 1.07)
**Dyspnoea (%)**	44 (86.3)	873 (89.5)	0.46	0.96 (0.86, 1.1)
**Hypercholesterolaemia (%)**	38 (74.5)	801 (82.2)	0.17	0.91 (0.77, 1.1)
**Hypertension (%)**	39 (76.5)	740 (75.9)	0.93	1.00 (0.86, 1.2)
**Type 2 Diabetes Mellitus (%)**	20 (39.2)	338 (34.7)	0.51	1.13 (0.80, 1.6)
**Heart Failure (%)**	15 (29.4)	91 (9.4) [*N* = 973]	< 0.001	3.15 (2.0, 5.0)
**Pulmonary Disease (%)**	4 (7.9)	149 (15.3)	0.15	0.51 (0.20, 1.3)
**Renal Disease (%)**	1 (2.0)	8 (0.8)	0.40	2.39 (0.31, 18.7)
**Neurological Disease (%)**	5 (9.8)	71 (7.3)	0.50	1.35 (0.57, 3.2)
**Extracardiac arteriopathy (%)**	5 (9.8)	145 (14.9)	0.32	0.66 (0.28, 1.5)
**Previous MI (%)**	46 (90.2)	616 (63.2)	< 0.001	1.43 (1.3, 1.6)
**Recent PCI (%)**	8 (15.7)	28 (2.9)	< 0.001	5.46 (2.6, 11.4)
**Clinically Unstable/Recent Deterioration (%)**	47 (92.2)	472 (48.4)	< 0.001	1.90 (1.7, 2.1)
**Preoperative inotropic support (%)**	4 (7.8)	5 (0.5)	< 0.001	15.3 (4.2, 55.2)
**Previous Cardiac Operation (%)**	0	4 (0.4)	0.65	
**Preoperative Hb (g/L) (± SD)**	132.8 (± 16.3)	139.3 (± 14.9)	0.003	
**Preoperative Creatinine (± SD)**	96.3 (± 62.8)	89.2 (± 34.8)	0.427	
**LVEF (%)** *Good* *Fair* *Poor*	23 (45.1) 16 (31.4) 12 (23.5)	723 (74.2) 221 (22.7) 31 (3.2)	< 0.001	
**Operative Priority (%)** *Elective* *Urgent* *Emergency* *Salvage*	3 (5.9) 33 (64.7) 13 (25.5) 2 (3.9)	462 (47.4) 505 (51.8) 7 (0.7) 1 (0.1)	< 0.001	
**Total Bypass Time in mins (± SD)**	95.7 (± 39.3)	93.8 (± 29.0)	0.36	
**Cross-Clamp Time in mins (± SD)**	59.2 (± 18.4)	61.9 (± 21.2)	0.66	
**Euroscore II (± SD)**	7.5 (± 4.2)	3.9 (± 2.5)	< 0.001	
**Propensity Score for Aprotinin (± SD)**	0.37 (± 0.35)	0.03 (± 0.07)	< 0.001	

### Preoperative patient characteristics

Many preoperative factors did not significantly differ between the aprotinin-treated patients and control patients (Table 2). However, the factors that displayed statistically significant differences could considerably affect postoperative outcomes. Aprotinin-treated patients were almost twice as likely to be clinically unstable, OR 1.9 [1.7, 2.1], and 15 times more likely to be on preoperative inotropic support, OR 15.3 [4.2, 55.2]. The aprotinin group also had higher rates of heart failure and worse left ventricular ejection fraction (LVEF) with an OR of 2.33 [0.98, 5.56]. These patients were also more likely to have had previous MI, OR 1.43 [1.3, 1.6], or recent percutaneous intervention (PCI), OR 5.46 [2.6, 11.4]. The mean European System for Cardiac Operative Risk Evaluation (EuroSCORE) score for the control group was 3.9 [± 2.5] which was almost double the score of the aprotinin cohort (7.5 [± 4.2]), indicating a significantly greater preoperative risk in these patients. Although propensity score matching reduced some of these differences, there remained statistically significant differences between the groups in terms of the incidence of heart failure, preoperative MI and poor LVEF. Furthermore, the mean EuroSCORE remained considerably greater in the aprotinin group (7.3 [± 4.0]) than in the control group (4.8 [± 3.9]) (Table 1).

**Table 2:** Statistically significant patient characteristics (propensity-matched cohort)

Baseline characteristics of propensity matched cohort				
Variable	Aprotinin (*N* = 48)	Control (*N* = 48)	*P* value	Odds ratio for aprotinin (95% CI)
**Heart Failure (%)**	14 (29.2)	6 (12.5)	0.04	2.33 (0.98, 5.56)
**Previous MI (%)**	43 (89.6)	34 (70.8)	0.02	1.27 (1.0, 1.6)
**Clinically Unstable/Recent Deterioration (%)**	44 (91.7)	27 (56.3)	< 0.001	1.63 (1.25, 2.12)
**LVEF (%)** *Good* *Fair* *Poor*	23 (47.9) 14 (29.2) 11 (22.9)	35 (72.9) 8 (16.7) 5 (10.4)	0.04	
**Operative Priority (%)** *Elective* *Urgent* *Emergency* *Salvage*	3 (6.3) 32 (66.7) 11 (22.9) 2 (4.2)	21 (43.8) 21 (43.8) 6 (12.5) 0	< 0.001	
**Euroscore II (± SD)**	7.3 (± 4.0)	4.8 (± 3.9)	0.003	
**Propensity Score for Aprotinin (± SD)**	0.33 (± 0.31)	0.12 (± 0.21)	< 0.001	

### Primary and secondary outcomes

Reports of the primary and secondary outcomes in the unmatched cohorts and propensity matched are displayed in Tables 3 and 4, respectively.

**Table 3:** Primary and secondary outcomes (Unmatched cohort)

Primary and secondary outcomes				
Variable	Aprotinin (*N* = 51)	Control (*N* = 975)	*P* value	Odds ratio for aprotinin (95% CI)
**In-Hospital Deaths (%)**	6 (11.8)	17 (1.7)	< 0.001	6.75 (2.8, 16.4)
**Postoperative Transfusion Received (%)**	17 (33.3)	258 (26.5)	0.33	1.26 (0.84, 1.9)
**Reopen (Bleed/tamponade) (%)**	4 (7.8)	31 (3.2)	0.09	2.47 (0.91, 6.7)
**Postoperative Myocardial Infarction (%)**	0	12 (1.2)	1.0	
**Postoperative Dialysis (%)**	3 (5.9)	10 (1.0)	0.02	5.74 (1.6, 20.2)
**Postoperative Stroke (%)**	2 (3.9)	9 (0.9)	0.1	4.24 (0.9, 19.2)
**ICU Stay in hours (± SD)**	69.1 (± 85.2)	50.3 (± 55.2)	0.13	
**Postoperative Stay in days (± SD)**	10.1 (± 10.7)	7.5 (± 6.5)	0.09	

**Table 4:** Primary and secondary outcomes (Propensity-matched cohort)

Outcomes in propensity matched cohort				
Variable	Aprotinin (*N* = 48)	Control (*N* = 48)	*P* value	Odds ratio for aprotinin (95% CI)
**In-Hospital Deaths (%)**	5 (10.4)	2 (4.2)	0.44	2.5 (0.51, 12.3)
**Postoperative Transfusion Received (%)**	16 (33.3)	10 (20.8)	0.25	1.6 (0.81, 3.2)
**Reopen (Bleed/tamponade) (%)**	4 (8.3)	2 (4.2)	0.68	2.0 (0.38, 10.4)
**Postoperative Dialysis (%)**	3 (6.3)	1 (2.1)	0.62	3.0 (0.32, 27.8)
**Postoperative Stroke (%)**	2 (4.2)	0	0.50	
**ICU Stay in hours (± SD)**	68.7 (± 86.4)	46.8 (± 38.5)	0.11	
**Postoperative Stay in days (± SD)**	10.4 (± 10.9)	7.5 (± 5.8)	0.11	

In the unmatched cohort, the mortality rate in the aprotinin cohort (11.8%) was significantly higher than in the control group (1.7%), OR 6.75 [2.8, 16.4] and p-value < 0.001. In terms of secondary outcomes, there were significantly more patients who required postoperative dialysis in the aprotinin group (5.9%) than in the control group (1.0%), OR 5.74 [1.6, 20.2] and *p* = 0.02. None of the other secondary outcomes reached statistical significance.

In the propensity score-adjusted cohort, a total of 7 deaths were reported, 5 (10.4%) in the aprotinin group and 2 (4.2%) in the control group (OR 2.5 [0.51, 12.3]), although this difference did not reach statistical significance (*p* = 0.44).

In terms of secondary outcomes, the aprotinin group had a greater incidence of postoperative renal failure requiring dialysis (6.3% in the aprotinin group vs. 2.1% in the control group, OR 3.0 [0.32, 27.8]) and stroke (4.2% in the aprotinin group vs. 0% in the control group). However, neither of these differences reaches statistical differences with p-values of 0.62 and 0.50, respectively. There was no instance of postoperative MI recorded in the propensity score-matched cohort. There was also a higher rate of transfusion in the aprotinin cohort (33.3%) than in the control group (20.8%), OR 1.6 [0.81, 3.2]. However, neither the rate of transfusion nor the number of units used was significantly different between the groups (*p* = 0.25 and *p* = 0.53, respectively). Patients receiving aprotinin had longer average ICU stay (68.7 ± 86.4 h) and total postoperative hospital stay (10.4 ± 10.9 days) than did those in the control group (46.8 ± 38.5 h and 7.5 ± 5.8 days, respectively).

## Discussion

This study reports a single-centre experience using aprotinin in a high-risk patient cohort undergoing iCABG surgery. The propensity score-matched analysis of 1026 patients, including 51 patients treated with aprotinin, revealed noteworthy findings. Propensity-score matching was not able to eliminate all baseline risk and comorbidity differences between the two cohorts. Despite significantly greater preoperative risks in the aprotinin group, such as increased rates of heart failure, poorer left ventricular ejection fraction, and higher EuroSCORE, there were no statistically significant differences in hospital deaths compared to the control group. Nevertheless, there was a 5.8% difference in the mortality rate between the aprotinin-treated patients and the control patients, which could indicate an element of clinical importance. Arguably, the sample size was too small to reliably determine the statistical significance of the differences.

A secondary analysis of the propensity score-matched data was also conducted to investigate the cause of mortality. The average EuroSCORE was 13 (range: 11–15) among the patients who died, which was significantly greater than that of the average cohort population. Furthermore, both instances of postoperative stroke and 2 out of 3 cases requiring new dialysis in the aprotinin cohort were among the patients who died before discharge. Perhaps even more significantly, surgery for 80% of patients who died in hospital were classified as emergency procedures.

The secondary outcomes of new renal replacement therapy and postoperative stroke were not significantly different, although they were more comparable between the two groups, and any difference could be due to worse preoperative conditions in the aprotinin group. Furthermore, concerns about poor renal outcomes and postoperative strokes may be partly linked to the nature and risk of surgery rather than exclusively the use of aprotinin. A large cohort study conducted by Furnary et al. suggested that poor renal outcomes were related to increased transfusions in high-risk patients, an established cause of renal dysfunction, and the absence of aprotinin use [13]. Furthermore, even in the BART study, aprotinin did not significantly affect the incidence of renal failure [9]. Although the aprotinin cohort had slightly greater rates of transfusion, this may be closely linked to the fact that most of these patients were urgent and did not stop DAPT or had platelet dysfunction prior to surgery.

The study also evaluated postoperative recovery periods between the two groups. These differences approached statistical significance, suggesting a possible trend toward longer recovery periods in the aprotinin group (*p* = 0.11 for ICU stay and *p* = 0.13 for hospital stay). However, since the baseline characteristics of the aprotinin cohort were worse than those of the control group, prolonged ICU and hospital stay may have been expected.

The reintroduction of aprotinin was associated with several conditions, including a limited licence in iCABG and a high risk of blood loss. Many studies have shown that aprotinin is safe for use in high-risk surgery. A large meta-analysis of more than 30,000 patients by Mehbohm et al. [14] showed that early mortality in high-risk patients did not differ between aprotinin and lysine analogue use. Other retrospective studies have also shown decreased mortality and reduced blood loss associated with aprotinin use in high-risk and open-heart procedures [15, 16]. Walkden et al. have also shown that since the withdrawal of aprotinin, mortality in high-risk cardiac surgery patients has significantly increased [17]. Furthermore, a large observational study conducted around the same time as the BART study showed that aprotinin did not adversely affect short- or medium-term survival [18]. These findings were further supported by a large-scale meta-analysis by Howell et al., which showed no increase in mortality with aprotinin compared to other antifibrinolytics [19]. According to Health Canada and the EMA, the therapeutic advantage of aprotinin may outweigh its risks in many cases and does not increase mortality [10, 11].

There are certain limitations to the studies since 2006, including the BART trial, which has been called into question regarding issues in the analysis, methods, and participant selection. Most of the studies were observational and included patients with a very high preoperative risk when aprotinin was used, increasing the bias against aprotinin when poor outcomes may have been the natural course due to the patients’ condition [20]. The BART trial also excluded iCABG patients and patients who underwent lower risk procedures. McMullan et al. mentioned unexplained exclusions of patients, which may have had statistically significant impacts on the results of the BART study [20].

Recently, the results from the analysis of the Nordic Aprotinin Patient Registry (NAPaR) have been discussed in the European Journal of Anaesthesia. The authors describe comparable outcomes between iCABG patients in the NAPaR cohort and those from the last 10 years. Notably, the baseline characteristics of the iCABG patients in the registry were more comparable to those of the patients in the control cohort of this study. Drawing from the conclusions of this study, NAPaR provides more concrete evidence regarding the lack of a causal link between aprotinin use and mortality as well as secondary outcomes [12]. However, the study did not use any matching or adjustment for baseline differences in patients before comparing outcomes. The addition of data to the NaPaR registry is key to improving the availability of evidence that allows for comprehensive evaluation of aprotinin in the future.

It is important to recognise that this is one of very few studies that has discussed the outcomes of aprotinin use since its reintroduction. The key difference in methodology is the use of logistic regression powered propensity score matching and the limitation of patients to iCABG surgery. This allows more relevant comparisons between the aprotinin and control groups in this cohort. Our results show statistically insignificant differences in the mortality and all other major postoperative complications unlike much of the evidence that was cited prior to aprotinin’s suspension. Most of these studies either showed increase in mortality or increased postoperative complications such as renal dysfunction or stroke [5–9].

The greatest limitation of the study is the small patient cohort with no medium-to-long-term data, which makes it difficult to assess mortality outcomes. Secondly, since this study was designed retrospectively after data collection, the paucity of reliable long-term data for most patients made it difficult to comment accurately on longer-term outcomes or use in survival analysis. Finally, although multivariable logistic regression-powered propensity matching was utilised to mitigate differences between the aprotinin-treated patients and control patients, the quality of the matching may not be ideal, as shown by the persisting risk difference in the matched cohorts. Unfortunately, the current practice of reserving aprotinin for use in high-risk patients makes mitigating this bias difficult [21].

The landscape of cardiac surgery is constantly changing. Even as early as 2005, research showed trends toward increasing rates of iCABG in high-risk patient groups. Patients who underwent surgery tended to be older and had more comorbidities [22]. This trend, as described by Dimeling G et al., has continued to increase in recent years [23]. More patients than ever are undergoing urgent surgical revascularisation and using dual antiplatelet therapies, leading to a higher risk of bleeding during surgery [24]. Moreover, surgical techniques and blood conservation strategies have made major leaps in the time since aprotinin was first suspended [25, 26]. This means that surgeons need reliable and contemporary evidence that supports the use of safe and effective antifibrinolytic therapy in high-risk surgery.

Further randomised trials with robust methods or a multicentre trial through analysis of a registry may allow for better mitigation of the baseline characteristics between patients. Future studies should also employ a power calculation to determine the minimum effect size. Future work should compare standardised, comparable patients who received aprotinin to those who did not, ideally through a large randomised controlled trial.

## Conclusion

This study included real-world outcome data on the use of aprotinin in high-risk patients. This is a preliminary study that shows comparable outcomes to control patients and suggests that aprotinin may be safely used in a select group of high-risk iCABG patients. The study highlights the ambivalence in existing literature and dearth of reliable evidence in this arena which should be addressed through adequately powered clinical trials in the future.

### Electronic supplementary material

Below is the link to the electronic supplementary material.

Supplementary Material 1

## Data Availability

The authors agree to share the data underlying the reported analyses by readers upon reasonable request. The authors would like to invite interested investigators to collaborate in conducting future research regarding the use of aprotinin in clinical practice.
