# Supplementary material for: Aprotinin in high-risk isolated coronary artery bypass graft patients: a 3-year propensity matched study
Source: J Cardiothorac Surg. 2024 Jul 18;19:459. doi: 10.1186/s13019-024-02837-1 (PMC11256430; doi:10.1186/s13019-024-02837-1)
Supplement: Supplementary file 1 — Supplementary Material 1 [file 13019_2024_2837_MOESM1_ESM.pdf]

Standard Operating Procedure for the Use of Aprotonin in Cardiac Surgery

Department of Cardiothoracic Surgery

Castle Hill Hospital

Hull University Teaching Hospitals NHS Trust

1. Introduction

1.1 An association between Aprotinin use and increased mortality was reported in some nonrandomized observational studies (eg, Mangano 2007, Schneeweiss 2008, Olenchock 2008, Shaw 2008) while other non-randomized studies have not reported such an association (eg, Karkouti 2006, Mangano 2006, Coleman 2007, Pagano 2008, Ngaage 2008, Karkouti, 2009). In these studies, Aprotinin was usually administered to patients who had more risk factors for increased mortality before surgery than patients in the other treatment groups.

1.2 Most of the studies did not adequately account for these baseline differences in risk factors and the influence of these risk factors on the results is not known. Therefore interpretation of these observational studies is limited and an association between aprotinin use and increased mortality can neither be established nor refuted. Thus, aprotinin should only be used as authorized in isolated CABG surgery, after careful consideration of the potential risks and benefits.

1.3 A publication by Fergusson et al 2008 analyzed data from a randomized controlled trial, Blood Conservation Using Antifibrinolytics in a Randomized Trial (BART), and reported a higher mortality rate in aprotinin-treated patients compared to those treated with tranexamic acid or aminocaproic acid. However, due to several methodological deficiencies no firm conclusion on cardiovascular risks can be made on the BART study results.

2. Approval for reintroduction

2.1 Approval for the reintroduction of Aprotonin for the reduction of bleeding and need for the use of blood products after cardiac surgery was awarded from the Drugs and Therapeutics Committee in March 2019 (appendix 1).

2.2 This is dependent on the introduction of SOP and participation in the Nordic Aprotinin Patient Registry (NAPaR). This is a requirement stipulated by the EMA and MHRA in accordance with the licence that centres can only be supplied with licensed Aprotinin from Nordic Pharma if they are signed up to an agreement to participate in the NAPaR.

3. Indications and Contraindications

3.1 Aprotonin was approved for the licensed indication, as stated on SmPC for adult patients who are at high risk of major blood loss undergoing isolated coronary artery bypass graft surgery (ie. coronary artery bypass surgery that is not combined with other cardiovascular surgery). Aprotinin should only be used after careful consideration of the benefits and risks, and consideration of alternative treatments available such as Tranexamic Acid.

3.2 Patient characteristics considered to increase the risk for perioperative bleeding*

3.2.1 Underlying comorbidities such as previous cardiac surgery or hepatic dysfunction affecting clotting
3.2.2 Inherited/acquired coagulation abnormalities
3.2.3 Low platelet count of less than 150
3.2.4 Platelet dysfunction
3.2.5 Dual antiplatelet therapy
3.2.6 New oral anticoagulants therapy (dabigatran, rivorixaban, apixaban)
3.2.7 Jehovah's witness/refusal of blood transfusion

**Aprotinin: is it time to reconsider? The European Society of Anaesthesiology task force reports on the place of aprotinin in clinical anaesthesia European Journal of Anaesthesiology: September 2015 - Volume 32 - Issue 9 - p 591–595.*

3.3 The following are indications for the use of Aprotinin:
3.3.1 **In emergency patients** (eg coronary dissection or occlusion in the Cath Lab or similar emergency) where patient had to be operated immediately.
3.3.2 **In urgent patients** where an inpatient CABG had to be performed earlier than after 5 days of stopping dual antiplatelet or anticoagulant therapy. The urgent nature for surgery needs to be justified and agreed between all members of team and the patient, for example, on-going chest pain or acute heart failure due to ongoing ischaemia and not responding to medical therapy.
3.3.3 **In selected elective patients** where there is higher risk of bleeding but there are no other alternatives. These are patients with congenital or acquired coagulopathy and where bleeding risk is significant despite being maximally treated on the advice of the Consultant Haematologists.

3.4 Contraindications for the use of Aprotinin:

3.4.1 There is no clear evidence for safety of Aprotinin in Mini CPB Circuit (MIECC) and therefore it should not be used in cases where mini CPB system is used.

3.4.2 Hypersensitivity to the active substance or any of the excipients: Sodium chloride, water for injections.

3.4.3 Patients with a positive aprotinin-specific IgG antibody test are at an increased risk of anaphylactic reaction when treated with aprotinin. Therefore, administration of aprotinin is contraindicated in these patients.

3.4.4 In case no aprotinin specific IgG antibody test is possible prior to treatment, administration of Aprotinin to patients with a suspected previous exposure to Aprotinin including fibrin sealant products (Tisseel and Artiss) during the last 12 months is contraindicated.

3.4.5 **Aprotinin should not be used when CABG surgery is combined with another cardiovascular surgery because the benefit risk balance of aprotinin in other cardiovascular procedures has not been established.**

3.5 The following is not a contraindication:

3.5.1 *Renal impairment*

Available clinical experience suggests that patients with decreased renal function do not require special dose adjustment.

3.5.2 Hepatic impairment

No data are available on dosage recommendations for patients with hepatic dysfunction.

3.5.3 *Elderly*

Reported clinical experience has not identified differences in responses in elderly patients.

4. Administration and intraoperative monitoring

4.1 The Consultant Surgeon and Anaesthetist have to agree that Aprotonin is indicated in the case being considered and this clearly stated at the Team Brief in the morning and the Perfusionist included in the discussion. It is preferable to have this discussion prior to the day of surgery especially and have it documented in the notes. This to be performed at pre-assessment for elective patients

4.2 It is essential to discuss the use of Aprotonin and its risks and benefits with the patient and have this documented in the notes.

4.3 Aprotonin will be prescribed by the Consultant Anaesthetist according to the following protocol:

4.3.1 Aprotinin 10,000 KIU/ml Injection. Each 50ml vial contains aprotinin solution corresponding to 500,000 Kallikrein Inactivator Units, KIU aprotinin in 0.9% sodium chloride solution.

4.3.2 An appropriate aprotinin-specific IgG antibody test may be considered before administration of Aprotinin.

4.3.3 Owing to the risk of allergic/anaphylactic reactions, a 1 ml (10,000 KIU) test dose should be administered to all patients at least 10 minutes prior to the remainder of the dose. After the uneventful administration of the 1 ml test dose, the therapeutic dose may be given.

4.3.4 A loading dose of 1 - 2 million KIU is administered as a slow intravenous injection or infusion over 20 - 30 minutes after induction of anaesthesia and prior to sternotomy. A further 1 - 2 million KIU should be added to the pump prime of the heart-lung machine. To avoid physical incompatibility of Aprotinin and heparin when adding to the pump prime solution, each agent must be added during recirculation of the pump prime to assure adequate dilution prior to admixture with the other component. In order for this to occur in Prime Displacement CPB, the Aprotinin will be administered into the venous line once prime displacement is completed and the patient has gone onto cardiopulmonary bypass.

4.3.5 The initial bolus infusion is followed by the administration of a continuous infusion of 250,000 - 500,000 KIU per hour until the end of the operation.

4.3.6 In general, the total amount of Aprotinin administered per treatment course should not exceed 7 million KIU.

4.3.7 Aprotinin should be infused using a central venous catheter. The same lumen should not be used for the administration of any other medicinal product. When using a multi-lumen central catheter a separate catheter is not required.

4.3.8 Aprotinin must be given only to patients in the supine position and must be given slowly (maximum 5 - 10 ml/min) as an intravenous injection or a short infusion

4.3.9 **In patients undergoing cardiopulmonary bypass with aprotinin therapy, Activated Clotting Time (ACT) should be used to monitor and maintain adequate anticoagulation, a minimal celite-ACT of 750 seconds, independent of the effects of haemodilution and hypothermia, is recommended in the presence of Aprotinin**.

4.3.10 In aprotinin treated patients the neutralisation of heparin by protamine after discontinuation of cardiopulmonary bypass should be based on a fixed ratio to the amount of heparin applied.

4.3.11 When the decision to use Aprotinin has been taken, the bleeding risk is considered to be significant, the case should not be a training case and is done by Consultant Surgeons and Anaesthetists.

5. Precautions

5.1 Graft Conservation: Blood drawn from the aprotinin central infusion line should not be used for graft preservation.

5.2 Re-exposure to aprotinin

Administration of aprotinin, especially to patients who have received Aprotinin (including aprotinin containing fibrin sealants) in the past requires a careful risk/benefit assessment because an allergic reaction may occur. Although the majority of cases of anaphylaxis occur upon re-exposure within the first 12 months, there are also single case reports of anaphylaxis occurring upon re-exposure after more than 12 months.

5.3 Aprotinin has a dose-dependent inhibitory effect on the action of thrombolytic agents, e.g. streptokinase, urokinase, alteplase (r-tPA).

5.4 Renal dysfunction could be triggered by Aprotinin, particularly in patients with pre-existing renal dysfunction. Aminoglycosides are a risk factor for renal dysfunction.

5.5 **Fertility, Pregnancy and Lactation**

5.5.1 Pregnancy

There are no adequate and well-controlled studies in pregnant women. Aprotinin should be used throughout pregnancy only if the potential benefit justifies the potential risk. In case of severe adverse drug reactions (like anaphylactic reaction, heart arrest, etc.) and their consecutive therapeutic measures, damage to the foetus has to be taken into account for a risk/benefit evaluation.

5.5.2 Breastfeeding

It is unknown whether aprotinin is excreted in human milk. However, since aprotinin is not bioavailable after oral administration, any drug contained in the milk is not expected to have a systemic effect on the breast-feed child.

5.5.3 Fertility

There are no adequate and well-controlled studies addressing fertility in men or women.

6. Potential Adverse Effects

6.1 Uncommon Side effects

6.1.1 Cardiac: Myocardial ischaemia, Coronary occlusion/ thrombosis, Myocardial infarction, Pericardial effusion

6.1.2 Vascular: Thrombosis

6.1.3 Renal: Oliguria, Acute renal failure, Renal tubular necrosis

6.2 Rare Side Effects

6.2.1 Immune System: Allergic reaction, Anaphylactic / anaphylactoid reaction

6.2.2 Vascular: Arterial thrombosis (and its organ specific manifestations that might occur in vital organs such as kidney, lung or brain)

6.3 Very Rare Side Effects

6.3.1 Immune System: Anaphylactic shock (potentially life threatening)

6.3.2 Blood and Lymphatic System: Disseminated intravascular coagulation/Coagulopathy

6.3.3 Vascular Disorder: Pulmonary embolism

6.3.4 General disorders or administration site conditions: Injection and infusion site reactions/

Infusion site (thrombo-) phlebitis

Frequencies are defined as:

Common: $\geq$ 1/100 to < 1/10

Uncommon: $\geq$ 1/1,000 to < 1/100

Rare: $\geq$ 1/10,000 to < 1/1,000

Very rare: < 1/10,000

7. Overdose

There is no specific antidote

8. Incompatibilities

Aprotinin is incompatible with antibiotics such as tetracyclines which react with proteins, corticosteroids, heparin and nutrient solutions containing amino acids or fat emulsions. The addition of aprotinin to mixed infusions (particularly with beta-lactam antibiotics) should be avoided. Electrolyte and sugar solutions are compatible with aprotinin.

9. Special Precautions for Storage

Store at room temperature (not more than 25°C); protect from light. Aprotonin will be stored in the Pharmacy Cupboard in the Cardiac Theatre and stocks monitored and maintained by Pharmacy Staff.

10.Registry and Governance

10.1 If Aprotonin is used this should be identified in the Patients Analysis and Tracking System (PATS) by the Surgeon completing the database

10.2 If Aprotonin is administered the data sheet of the **NORDIC APROTININ PaTIENT REGISTRY (NAPaR)** (Appedix II) will be completed by the Anaesthetist and this is placed in the patients notes. Neil Richards will enter the data into the registry electronically.

10.3 All cases where Aprotonin is used should be presented at the monthly Morbidity and Mortality Meeting.

Patient undergoing Isolated Coronary Artery Bypass Surgery

Yes

NO

No Aprotonin

High Risk of Bleeding:

Underlying comorbidities

Inherited/acquired coagulation abnormalities

Low platelet count

Platelet dysfunction

Dual antiplatelet therapy

New oral anticoagulants (dabigatran, rivorixaban, apixaban)

Jehovah's witness/refusal of blood transfusion

No Contraindication such as:

Positive Aprotonin Specific IgG antibody

Aprotonin or Fibrin Sealents used in the last 12 months

*No safety evidence in Mini CPB Circuit therfore Aprotonin should not be used

Clear Discussion and Joint decision between Surgeon and Anaesthetist and appropriate documentation in the notes

Discussion of Risk and benefit with the patient and documentation in the notes

Adminster Aprotonin as per SOP:

-1 ml (10,000 KIU) test dose should be administered to all patients at least 10 minutes prior to the remainder of the dose

- A loading dose of 1 - 2 million KIU is administered as a slow intravenous injection or infusion over 20 - 30 minutes after induction of anaesthesia and prior to sternotomy.

- A further 1 - 2 million KIU should be added to the CPB machine.

Anaethetist: Complete Registry paper form and place it in the notes

Surgeon: Tick the Aprotonin used box in the PATS
